# Senescence-Associated Secretory Phenotype Suppression Mediated by Small-Sized Mesenchymal Stem Cells Delays Cellular Senescence through TLR2 and TLR5 Signaling

**DOI:** 10.3390/cells10010063

**Published:** 2021-01-03

**Authors:** Ji Hye Kwon, Miyeon Kim, Soyoun Um, Hyang Ju Lee, Yun Kyung Bae, Soo Jin Choi, Hyun Ho Hwang, Wonil Oh, Hye Jin Jin

**Affiliations:** 1 Biomedical Research Institute, MEDIPOST Co., Ltd., Seongnam 13494, Korea; kjh0127@medi-post.co.kr (J.H.K.); eldjfls3@medi-post.co.kr (M.K.); ssoso23@medi-post.co.kr (S.U.); hjlee@medi-post.co.kr (H.J.L.); byk819@medi-post.co.kr (Y.K.B.); sjchoi@medi-post.co.kr (S.J.C.); wioh@medi-post.co.kr (W.O.); 2 King Abdullah University of Science and Technology, Thuwal 47000, Makkah Province, Saudi Arabia; heno.hwang@kaust.edu.sa

**Keywords:** small cell, mesenchymal stem cell senescence, senescence-associated secretory phenotype, growth-regulated oncogene-alpha, interukin-8, C-X-C motif chemokine receptor 2, toll-like receptor 2, toll-like receptor 5, cell-based therapy

## Abstract

In order to provide a sufficient number of cells for clinical use, mesenchymal stem cells (MSCs) must be cultured for long-term expansion, which inevitably triggers cellular senescence. Although the small size of MSCs is known as a critical determinant of their fate, the main regulators of stem cell senescence and the underlying signaling have not been addressed. Umbilical cord blood-derived MSCs (UCB-MSCs) were obtained using size-isolation methods and then cultured with control or small cells to investigate the major factors that modulate MSC senescence. Cytokine array data suggested that the secretion of interukin-8 (IL-8) or growth-regulated oncogene-alpha (GROa) by senescent cells was markedly inhibited during incubation of small cells along with suppression of cognate receptor (C-X-C motif chemokine receptor2, CXCR2) via blockade of the autocrine/paracrine positive loop. Moreover, signaling via toll-like receptor 2 (TLR2) and TLR5, both pattern recognition receptors, drove cellular senescence of MSCs, but was inhibited in small cells. The activation of TLRs (2 and 5) through ligand treatment induced a senescent phenotype in small cells. Collectively, our data suggest that small cell from UCB-MSCs exhibit delayed cellular senescence by inhibiting the process of TLR signaling-mediated senescence-associated secretory phenotype (SASP) activation.

## 1. Introduction

Multipotent mesenchymal stem cells (MSCs) can self-renew and secrete various trophic factors [1]. Among MSCs from different sources, umbilical cord blood-derived MSCs (UCB-MSCs) are easy to isolate, have a high proliferation rate, and express various beneficial paracrine factors [2]. In addition, the low immunogenicity and excellent immunomodulatory properties of UCB-MSCs lower the risk of immunological rejection during allogeneic transplantation [2].

MSCs have the ability to migrate to injury sites and suppress immune responses, highlighting the immense potential of these multipotent cells in human regenerative medicine [3,4]. Initially, replacement of cells at the damaged sites with transplanted cells was thought to be the major mechanism of action of MSCs. However, studies have shown that MSCs mainly secrete factors that drive the regenerative process in damaged tissues, in turn promoting angiogenesis and regulating the immune system. Therefore, these paracrine effects constitute the major mechanism of action of MSC treatment [2,5,6].

The characteristics of MSCs allow for their wide use in clinical settings, and thus MSCs have been extensively used in treatment [7,8]. However, the clinical use of MSCs requires expansion in culture to obtain a large number of cells, which often leads to cellular senescence [9,10,11]. Furthermore, the rate of senescence is different for each cell, as MSCs are a heterogenous population, and, thus, pre-senescent cells need to be removed prior to clinical use [12,13,14]. MSCs are a powerful tool in regenerative medicine and are used for the unique cell-based treatment of various diseases, such as inflammation disease or age-related disease [15,16,17]. The administration of senescent MSCs leads to inefficient cell differentiation in clinical settings [14]. Therefore, studies on the process of senescence and the development of methods for detecting senescent cells are essential. To date, numerous studies have been performed on the various aspects of senescence. For example, most cellular stress that causes senescence activates DNA damage response (DDR) kinases including Ataxia Telangiectasia mutated (ATM) and Ataxia Telangiectasia and Rad3 related (ATR), which regulate p53 and p16 primarily through proteasomal and epigenetic regulation [18,19]. Recently, studies have demonstrated that senescent cells exhibit a senescence-associated secretory phenotype (SASP) or senescence-messaging secretion (SMS), whereby they secrete specific inflammatory factors, growth factors, and proteolytic enzymes [20,21]. Additionally, studies on SASP have suggested that changes in senescence-related DNA methylation patterns and histone modifications occur when MSCs undergo senescence [22]. In fact, DNA methyltransferase inhibitors and histone deacetylase inhibitors were shown to partially improve senescence in MSCs [22]. Senescent MSCs secrete excessive amounts of interleukin (IL)-6, IL-8, interferon (IFN)-ß or IFN-ג, monocyte chemoattractant protein-1 (MCP-1), matrix metalloproteinase-2 (MMP-2), and tissue inhibitor matrix -2 (TIMP-2), which reduce the immunomodulatory capacity and promote carcinogenesis in MSCs [23]. Furthermore, senescent MSCs continuously induce the production of inflammatory cytokines through the activation of toll-like receptor (TLR) signaling [24,25].

To delay senescence, small-sized cells were isolated, and their stem cell properties were assessed [26]. Also, cellular senescence was delayed in small cells [26]. Moreover, compared with the control group (large or heterogenous cell), the small cell showed better therapeutic function, such as stemness, differentiation potential, and reducing immune reactions on damaged lung [26]. Currently, studies on the molecular regulation of cellular senescence delay in small cells are lacking. Herein, we hypothesized that SASP secretion and upstream signaling, such as through TLRs, are downregulated in small cells, contributing to their delayed senescence when compared to heterogenous MSCs.

To assess the mechanism of delayed MSCs senescence in small cells during cell culture, arrays of inflammatory cytokines secreted from heterogenous and small cells were compared, and senescence-inducing proteins such as growth-regulated oncogene-alpha (GROa) and IL-8 were assessed. Moreover, we observed a positive loop between ligands GROa and IL-8 mediated via their receptor C-X-C motif chemokine receptor 2 (CXCR2) and driven by TLR2 and TLR5 signaling. Importantly, senescence-inducing signaling was active at a low level in small-sized MSCs, leading to delayed cellular senescence.

## 2. Materials and Methods

### 2.1. Cell Culture and Reagents

This study was approved by the Institutional Review Board of MEDIPOST Co., Ltd. (MP-2014-07-1-1). UCB samples were obtained from the umbilical vein after delivery of the neonates. Mononuclear cells (MNCs) were separated from UCB using Ficoll–Hypaque solution (density = 1.077 g/cm^3^; GE Healthcare, Uppsala, Sweden). MNCs were washed and suspended in Minimum Essential Medium *α* (Gibco/Invitrogen, Carlsbad, Grand Island, NY, USA) supplemented with 10% fetal bovine serum (Gibco). UCB-MSCs were cultured at 37 °C in a humidified atmosphere containing 5% CO_2_, and the culture medium was changed twice a week [27]. Each UCB-MSC from three donors was used in our study (*n* = 3). Detailed information related to the UCB-MSCs is summarized in Supplemental Table S1. Results showed that Pam3CSK4 and flagellin can induce TLR2 and TLR5 signaling pathway [28]. The pam3CSK4 and flagellin was obtained from Sigma-Aldrich (St. Louis, MO, USA). Recombinant human GROa or IL-8 were purchased from R&D Systems (Minneapolis, MN, USA). The anti-TLR2 and anti-TLR5 antibodies used to block TLR2 and TLR5 stimulation, respectively, were purchased from Abcam (Cambridge, UK).

### 2.2. Cell Growth Kinetics and Senescence Senescence-Associated Beta-Galactosidase (SA β-Gal) Staining

For growth kinetics, the expansion of cells was tested using the trypan blue exclusion method. At each passage, MSCs were cultured for 5–7 days, harvested with trypsin- Ethylenediaminetetraacetic acid (EDTA) (Gibco/Invitrogen, Carlsbad, Grand Island, NY, USA), counted, and then reseeded at a density of 500–2000 cells/cm^2^. The population doubling (PD) was analyzed for passages by dividing the logarithm of the 2 [10]. PD and cumulative PD measurements were repeated until the cells stopped proliferating. Senescence-associated beta-galactosidase (SA β-gal) staining was measured as a biomarker of senescence in UCB-MSCs. A Histochemical Staining Kit (Cell Signaling Technology, Danvers, MA, USA) was used according to the manufacturer’s instructions in order to determine SA β-gal activity, and the cells were examined using an inverted microscope (3 fields/cell were assessed). The percentage of senescent cells was represented by the number of stained cells to the total number of cells.

### 2.3. Umbilical Cord Blood-Derived Mesenchymal Stem Cells (UCB-MSC) Characterization

To analyze cell surface marker expression, cells were stained for 15 min at room temperature (25 °C) using fluorescein isothiocyanate (FITC)-conjugated antibodies against human CD14 and CD45 (BD Biosciences, Franklin Lakes, NJ, USA), as well as phycoerythrin (PE)-conjugated antibodies against human CD29, CD73, CD90, CD166, (BD Biosciences), and CD105 (Invitrogen). Corresponding isotype-matched mouse antibodies were used as controls. The cells were washed with phosphate-buffered saline (Gibco) and fixed with 1% (*v*/*v*) paraformaldehyde (Sigma-Aldrich). UCB-MSCs were analyzed using flow cytometry and a FACSCalibur instrument, and the percentage of cells expressing surface antigens was calculated for 10,000 gated-cell events. To assess multi-lineage potential, cells were cultured in specific media to induce their differentiation into osteocytes, chondrocytes, and adipocytes. After induction, multi-lineage potential was evaluated as previously reported [11]. Briefly, osteocyte formation was assessed by measuring the level of alkaline phosphatase using staining (Sigma-Aldrich), chondrocyte formation was determined using safranin O staining (Sigma-Aldrich), and adipocyte formation was assessed based on the staining of the accumulated lipid vacuoles using Oil Red O (Sigma-Aldrich).

### 2.4. Isolation by Cell Size

UCB-MSCs were separated into size groups by 10 µm diameter at P2, namely, a non-sieved population (control or heterogenous) and a population with diameters ≤10 µm (small). Size filtering was carried out using the subsequently described methods. For size, isolation we prepared the filter considering the risk of damaging MSCs as well as safety in usage. We used a Pluristrainer filtration membrane tube (10 µm pore size, pluriSelect, San Diego, CA, USA). (i) Filter mounting: the filtration membrane was inserted in 50-mL culture tubes. (ii) Cell loading: MSCs (1~2 × 10^5^/2 mL) were loaded on a filtration membrane tube. (iii) Obtaining two populations: non-filtered heterogenous cells and filtered small cells was obtained (Figure S1). For the measurement of cell size, cells were harvested, pelleted, suspended in media, and pipetted in a hemocytometer. Images were then acquired at 100× magnification (Olympus BX40, Center Valley, PA, USA). The cell diameter and circularity were measured using SABIA (MeTooSoft, Seoul, Korea). This process was demonstrated to enrich small cells without significant contamination. Small UCB-MSCs is summarized in Supplemental Table S1.

### 2.5. Cytokine Array and Enzyme-Linked Immunosorbent Assay (ELISA)

We collected conditioned media of the two groups after 24 h of incubation at P13 (heterogeneous cells vs. small cells). The presence of cytokines within the harvested conditioned medium was detected using a cytokine array membrane, namely the human cytokine array panel A, which contains 40 antibodies (R&D system, Supplemental Table S2). After subtracting background signals and normalizing values to the reference spots, signal intensities between and among array images were compared to determine the relative differences in expression levels of each protein between the two groups. Culture supernatants were collected, and IL-8 or GROa levels were measured using enzyme-linked immunosorbent assay (ELISA, R&D Systems) according to the manufacturer’s protocol.

### 2.6. Western Blotting

Cell extracts were prepared in a buffer containing 9.8 M urea, 4% CHAPS, 130 mM dithiothreitol, 40 mM Tris-HCl, and 0.1% sodium dodecyl sulfate. Protein concentrations were measured using a bicinchoninic acid assay kit (Sigma-Aldrich). Protein extracts (10 μg) were separated using SDS-polyacrylamide gel electrophoresis, and the resolved proteins were transferred onto nitrocellulose membranes. Each membrane was incubated with antibodies against phospho-p53 (P-p53), phospho-p38 (P-p38), p21, p16, and β-actin (Sigma-Aldrich).

### 2.7. Quantitative Polymerase Chain Reaction (qPCR) and Small Interfering RNA Experiments

Quantitative polymerase chain reaction (qPCR) was performed using a LightCycler^TM^ 480 (Roche, Mannheim, Germany). TaqMan probes were designed with the Universal Probe Library Assay Design Center and used to quantitatively detect mRNA transcript levels for genes encoding the following: IL-6, IL-8, IL-23, GROa, migration inhibitory factor (MIF-1), MCP-1, CXCR2, TLR (1–9), and *β*-actin. Relative expression levels of the mRNAs of interest were calculated using the comparative threshold cycle method (2^−^^ΔΔCt^) with normalization to *β*-actin mRNA expression [29]. siRNA against GROa, IL-8, CXCR2, as well as scrambled control siRNA were purchased from Dharmacon (Chicago, IL, USA). siRNA was transfected using Dharmafect Reagent (Dharmacon) according to the manufacturer’s instructions. The siRNA pools consisted of four different siRNA duplexes (Supplemental Table S3).

### 2.8. Statistical Analysis

Data were analyzed using GraphPad Prism 6.0 (GraphPad Software, version 6, La Jolla, CA, USA). All data are presented as the mean ± standard deviation (SD). Statistical differences were measured using one-way analysis of variance (ANOVA) followed by Fisher’s least significant difference (LSD) and unpaired *t*-test. A *p*-value < 0.05 was considered statistically significant.

## 3. Results

### 3.1. Small Cells from UCB-MSCs Exhibit Delayed Cellular Senescence Compared to Heterogeneous Cells

**Figure 1:**
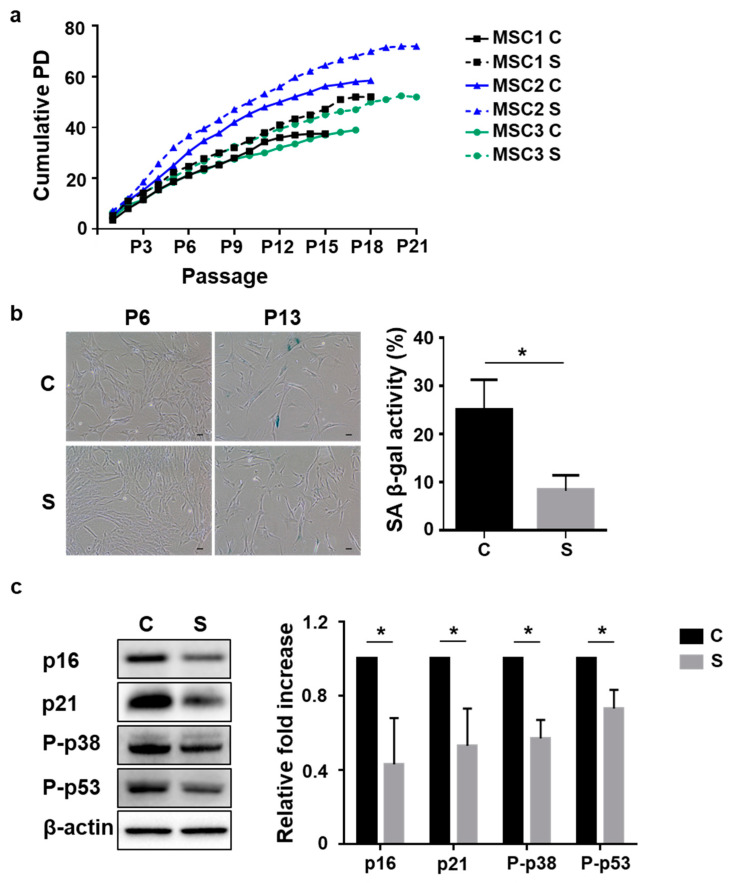
Small-sized cells from umbilical cord blood-derived mesenchymal stem cells (UCB-MSCs) exhibit delayed cellular senescence compared to heterogeneous cells during in vitro expansion. Three lots of UCB-MSCs separated by cell size isolation were cultured until cell growth stopped. (C: control or heterogenous cells, S: small-size cells); (**a**) the cumulative population doubling (PD) values were monitored until growth ceased; (**b**) the senescence activity was measured and quantified using senescence-associated beta-galactosidase (SA β-gal) staining at P6 and P13. SA β-gal staining revealed almost no positive cells in both groups at P6. Staining level increased considerably in MSCs in both groups from P6 to P13. Scale bars = 10 μm (left panel). SA β-gal activity was significantly lower in small cells compared to heterogenous cells at P13 (right panel); (**c**) expression levels of senescence-related proteins were analyzed at P13 using immunoblotting (left panel). The levels were normalized to those of ß-actin, with the expression level in control cells defined as 1 (right panel); (**b**,**c**) data are presented as mean ± standard deviation (SD), *n* = 3 per group. * *p* < 0.05. C, control cells; S, small cells; PD, population doubling; P, passage; SA ß-gal staining, senescence-associated beta-galactosidase staining; P-p53, phosphorylated p53; P-p38, phosphorylated p38.

Previous studies report that small-sized cells of MSCs have higher growth potential and a lower rate of senescence [26]. Herein, to better understand the senescence pathway in relation to small size, a comparative analysis was performed in small cells from UCB-MSCs isolated by filtering methods (Figure S1). We investigated the proliferative ability and senescence phenotype of control (non-isolated heterogeneous cells) and small cells from three different donors. The cell expansion potential was determined by counting cells at every passage (P) and was continuously monitored in culture flasks at regular time periods until cell growth stopped. As expected, small cells ceased to grow later and exhibited a senescence pattern with a higher cumulative population doubling (PD) over 18 passages when compared to control cells (Figure 1a). To evaluate various characteristics of cellular senescence, we analyzed SA β-gal staining and senescence-related protein levels. No positive staining for SA β-gal was observed in all populations until P6. In addition, no notable difference was observed in the morphology between the two groups at P6. SA β-gal-positive cells were considerably greater in both groups by P13. However, SA β-gal activity in small cells (8 ± 3%) was significantly lower than that in control cells (25 ± 6%). In both groups, cells became flattened and enlarged, especially the control cells, most of which exhibited these morphological changes (Figure 1b). In parallel, Western blot analysis revealed enhanced expression of p16, p21, phosphorylated p38 (P-p38), and phosphorylated p53 (P-p53) in control cells when compared to small cells at P13 (Figure 1c). Collectively, these data demonstrated that small cells from UCB-MSCs had greater proliferative ability and exhibited lower cellular senescence than heterogeneous cells.

### 3.2. Lower Levels of Senescence-Associated Secretion of Growth-Regulated Oncogene-Alpha (GROɑ) and Interleukin-8 (IL-8) by Small Cells

**Figure 2:**
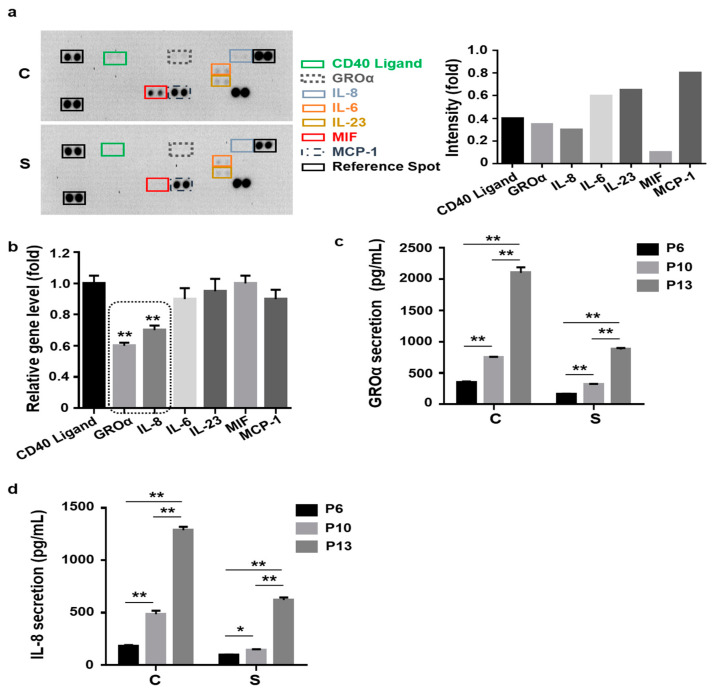
Inflammatory cytokine profile of UCB-MSCs with altered secretion following in vitro expansion. Both control and small MSCs were cultured until P13 under the same culture conditions: (**a**) Cytokine array analysis using cultured medium collected from control and small cells at P13. Secreted proteins were characterized using a cytokine array (40 spots, left panel). Seven spots in the membrane showing decreases in the small cell group are marked with squares (CD40 ligand, GROa, IL8, IL-6, IL-23, MIF, MCP-1). Quantification of the optical intensity for seven factors. Array analysis showing downregulated inflammatory cytokines in small cells compared to levels in control cells. Data normalized to the intensity of control cells, which was defined as 1 (right panel); (**b**) expression of the seven respective genes was quantified using quantitative polymerase chain reaction (qPCR). The expression levels of all genes were normalized to those of β-actin. Data normalized to the intensity of control cells, which was defined as 1. GROa and IL-8 exhibited the most significant decrease in small cells (black box); (**c**,**d**) To confirm the downregulation of GROa (**c**) and IL-8 (**d**), their secreted levels were measured in three different samples using enzyme-linked immunosorbent assay (ELISA) for both control and small cells at different passages; (**b**–**d**) data are presented as the mean ± standard deviation (SD), *n* = 3 per group. * *p* < 0.05, ** *p* < 0.01. C, control cells; S, small cells; P, passage; GROa, growth-regulated oncogene-alpha; IL-8, interleukin 8; MIF, macrophage migration inhibitory factor; MCP-1, monocyte chemoattractant protein-1; IL-6, interleukin-6; IL-23, interleukin-23.

**Figure 3:**
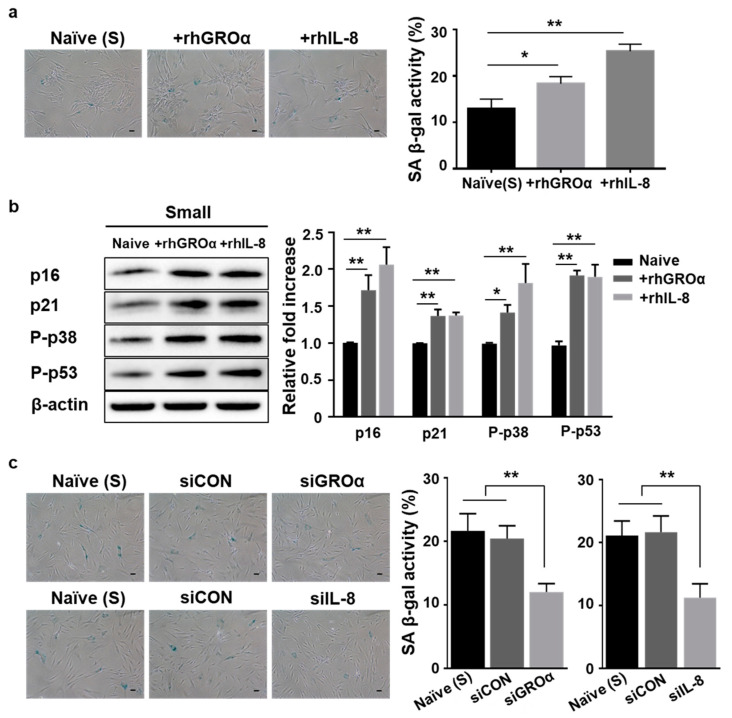
Both GROa and IL-8 induced the senescent phenotype in small cells isolated from UCB-MSCs: (**a**,**b**) Small cells at P13 were treated with recombinant human protein (GROa or IL-8) for 24 h. (**a**) Cells were stained to measure SA β-gal activity (left panel), and quantitation was performed by determining the percentage of SA β-gal-positive cells (right panel); (**b**) senescence-related proteins were analyzed using immunoblotting (left panel). Expression levels were normalized to β-actin, with the expression level in naïve cells defined as 1 (right panel); (**c**) small cells were transfected with scramble siRNA (siCon), GROa siRNA (siGROa), or IL-8 siRNA (siIL-8). The cells were stained with SA β-gal (left panel), and activity was measured by counting positively stained cells at P13 (right panel); (**a**,**c**) scale bars = 10 μm. Data are presented as mean ± SD, *n* = 3 per group. * *p* < 0.05. ** *p* < 0.01. C, control cells; S, small cells; P, passage; SA β-gal staining; senescence-associated β-galactosidase staining; rhGROa, recombinant human GROa; rhIL-8, recombinant human IL-8.

Senescent cells exhibit a characteristic protein secretion profile, also known as their “secretome” [20,21]. It has been reported that senescent MSCs express increased amounts of inflammatory cytokines, chemokines, and their receptors [23,30]. Thus, we hypothesized that small cells may utilize the SASP to actively control cellular senescence. To confirm SASP-related expression in control and small cells, we employed an inflammatory cytokine array containing 40 spots using culture medium at P13. As a result, using intensity analysis, we selected seven secretory proteins that exhibited low secretion in small cells, namely GROa, IL-8, CD40 ligand, macrophage migration inhibitory factor (MIF), MCP-1, IL-6, and IL-23 (Figure 2a). To confirm array data, we analyzed the expression levels of the seven respective genes in both groups using quantitative real-time PCR (qPCR). The expression levels of the five genes (CD40, IL-6, IL-23, MIF, and MCP-1) were not significantly different between the two groups. Notably, both GROa and IL-8 secretion were significantly low in small cells (Figure 2b). To further examine the association between secretion (GROa or IL-8) and cell senescence, we measured the amount of these two proteins in cultured media from the two groups (control vs. small cells) during expansion at P6, P10, and P13. The secretion of GROa or IL-8 gradually increased with subsequent passages in both groups (Figure 2c,d). Interestingly, cells of the two groups could be distinguished based on the concentration and rate of increase in GROa and IL-8 secretion with passaging. The increase in SASP-related secretion (GROa and IL-8) with cell passaging was markedly lower in small cells (Figure 2c,d). Groups exhibited significant differences in basal secretion (GROa: Control vs. Small; 348 pg/mL vs. 161 pg/mL; *p* < 0.05, IL-8: Control vs. Small; 181 pg/mL vs. 95 pg/mL; *p* < 0.05) at early P6 as well as in the increase from P6 to late P13 (GROa; Control vs. Small; 6.9-fold vs. 4.9-fold; *p* < 0.01, IL-8; Control vs. Small; 7.3-fold vs. 5.7-fold, *p* < 0.05, Figure 2c). Thus, these data indicated that the cellular expansion of MSCs promoted the SASP, enhancing both GROa and IL-8 secretion and determining the lesser degree of senescence observed in small cells. To identify the causative factor of the two proteins in relation to MSC cellular senescence, recombinant human (rh) GROa- or IL-8-treated control and small cells at P13 were assessed. Compared to untreated cells, GROa (100 ng/mL) and IL-8 (200 ng/mL)-treated cells exhibited significantly upregulated SA β-gal activity in both groups (Figure 3a and Figure S2). In small cells, both protein treatments significantly increased P-p38, P-p53, p21, and p16 levels (Figure 3b). To confirm whether the two secretory proteins functionally contributed to the cellular senescence of small cells, we inhibited GROa or IL-8 expression using siRNA. Control experiments demonstrated that treatment with target siRNA significantly inhibited GROa or IL-8 expression, as shown using qPCR (Figure S3a,b). Cells transfected with scramble siRNA (siCon) or siRNA against GROa and IL-8 were evaluated for the senescent phenotype. SA β-gal activity was effectively downregulated in GROa siRNA- and IL-8 siRNA-transfected cells (Figure 3c). Taken together, these findings indicated that inhibiting major SASP proteins (GROa and IL-8) accelerated cellular senescence in small cells from MSCs.

### 3.3. Positive Loop of GROa and IL-8 Secretion during MSC Senescence Is Controlled by Their Cognate Receptor C-X-C Motif Chemokine Receptor2 (CXCR2)

**Figure 4:**
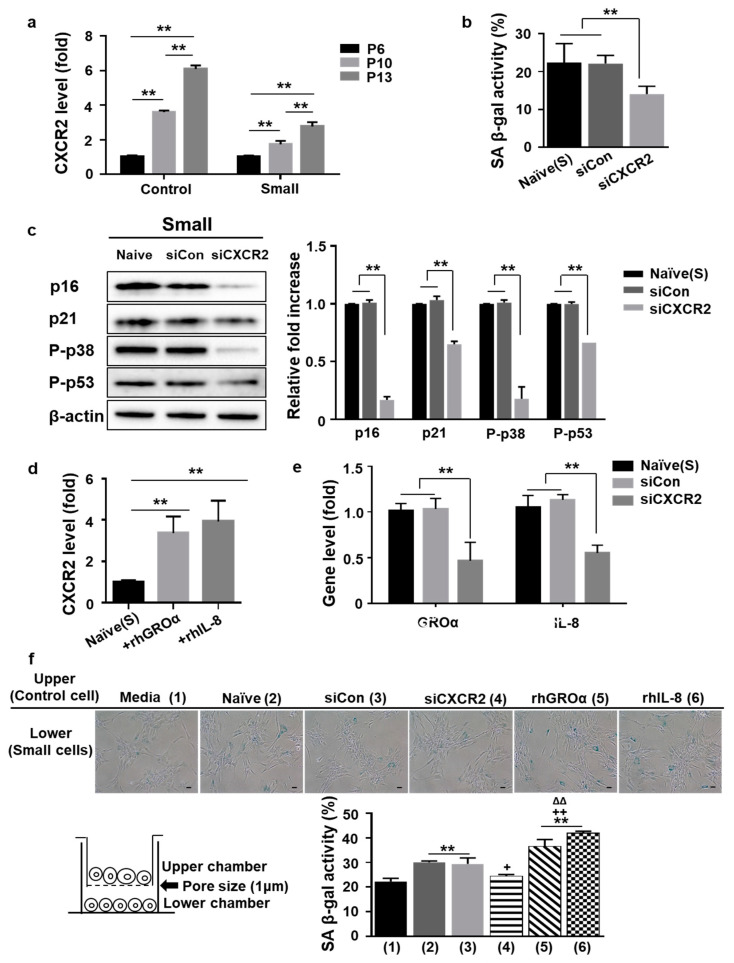
Autocrine/paracrine positive feedback between senescence-associated secretory phenotype (SASP)-associated protein (GROa and IL-8) and CXCR2 in UCB-MSC: (**a**) to confirm the increase of CXCR2 expression, gene expression was measured in three different samples using qPCR for both groups (control vs. small cells) at various passages following expansion. The level was normalized to β-actin, with expression at P6 defined as 1; (**b**) small cells were transfected with scramble siRNA (siCon) or CXCR siRNA (siCXCR2) at P13. The cells were stained to measure SA β-gal activity, and quantification was performed by determining the percentage of SA β-gal-positive cells. (**a**,**b**) Data are presented as mean ± SD, *n* = 3 per group. ** *p* < 0.01; (**c**) Western blot analysis of senescence-related proteins in small cells treated with CXCR2 siRNA (left panel). Protein levels were normalized to β-actin, with the expression level in naïve cells defined as 1 (right panel, data are presented as mean ± SD, *n* = 3 per group. ** *p* < 0.01); (**d**) small cells at P13 were treated with recombinant human protein (GROa or IL-8) for 24 h. The protein levels of CXCR2 were normalized to those of ß-actin. Data were normalized to the intensity of naïve cells, which was defined as 1. Data are presented as mean ± SD, *n* = 3 per group. ** *p* < 0.01; (**e**) small cells were transfected with scramble siRNA (siCon) or CXCR siRNA (siCXCR2). Expression of GROa or IL-8 was examined following depletion of CCR2 by knockdown. Gene expression levels were normalized to those of β-actin, with the expression level in the naïve cells defined as 1. Data are presented as mean ± SD, *n* = 3 per group. ** *p* < 0.01; (**f**) quantification of SA β-gal staining in small cells (lower chamber) after co-culture with control cells cultured in the upper chamber under various conditions including normal medium and cells ( naïve, siCon, siCXCR, rhGROa, or rhIL-8 treatment.) A transwell chamber was used that prevented direct cell contact owing to its pore size (1 µm, scheme). SA β-gal activity in small cells in the lower chamber was evaluated. Data are presented as mean ± SD, *n* = 3 per group. ** *p* < 0.01 vs. (1), + *p* < 0.05, ++ *p* < 0.01 vs. (2) or (3). ∆∆ *p* < 0.01 vs. (4). C, control cells; S, small cells; P, passage; SA β-gal staining; senescence-associated β-galactosidase staining; rhGROa, recombinant human GROa; rhIL-8, recombinant human IL-8.

Both GROa and IL-8 bind to their canonical receptor CXCR2, expressed on various immune, endothelial, and tumor cells, as well as MSCs [31]. Herein, the gene expression of CXCR2 in MSCs was determined using qPCR of the control and small cells obtained during expansion at P6, P10, and P13. CXCR2 expression was considerably upregulated from P6 to P13 in both groups. However, the groups exhibited significant differences between the respective increases from P6 and P13 (Control vs. Small: 6.2-fold vs. 2.6-fold; *p* < 0.01, Figure 4a). Moreover, siRNA-mediated silencing of CXCR2 in small cells remarkably inhibited cellular senescence, as indicated by SA β-gal activity (Figure S4 and Figure 4b) and the levels of senescence-related signaling proteins (P-p38, P-p53, p21, p16, Figure 4c). We confirmed that CXCR2 gene expression was markedly inhibited by siCXCR2 treatment (Figure S3c). These data indicated that CXCR2 is the main factor for the senescent phenotype of MSCs, as inhibiting CXCR2 accelerated cellular senescence in small cells. SASP was maintained by an autocrine/paracrine positive regulatory loop during cellular senescence [23]. To analyze whether the two secreted proteins (GROa and IL-8) could activate a similar signaling pathway through CXCR2, we investigated whether they could induce receptor expression. Stimulation of small cells with rhGROa or rhIL-8 protein significantly promoted the expression of CXCR2 in P13 (Figure 4d). In addition, the inhibition of CXCR2 by siRNA significantly blocked the expression of GROa or IL-8 compared to that in the control group (naïve or siCon), suggesting autocrine signaling via the two secreted factors and CXCR2 in control or small cells (Figure 4e and Figure S4). To determine whether GROa, IL-8, and CXCR2 could induce cellular senescence via paracrine activity, we co-cultured control and small cells at P13 in a transwell chamber that blocked the direct interaction of cells owing to its pore size (1 µm) (Figure 4f, scheme). The amount of SA β-gal-positive cells among small cells (low chamber) was altered by co-culture with control cells (senescent cells, upper chamber) when compared to those cultured using media alone. Importantly, treatment with rhGROa or rhIL-8 significantly increased SA β-gal activity. However, when control cells in the upper chamber were treated with CXCR2 siRNA, SA β-gal activity was significantly lower (Figure 4f). Taken together, these data indicated that the autocrine/paracrine feedback between the main SASP-associated proteins (GROa or IL-8) and CXCR2 is a key process in MSC senescence, and that small cells suppress this signaling.

### 3.4. Toll-Like Receptor 2 (TLR2)- and TLR5-Driven Cellular Senescence Was Inhibited in Small Cells from MSCs

**Figure 5:**
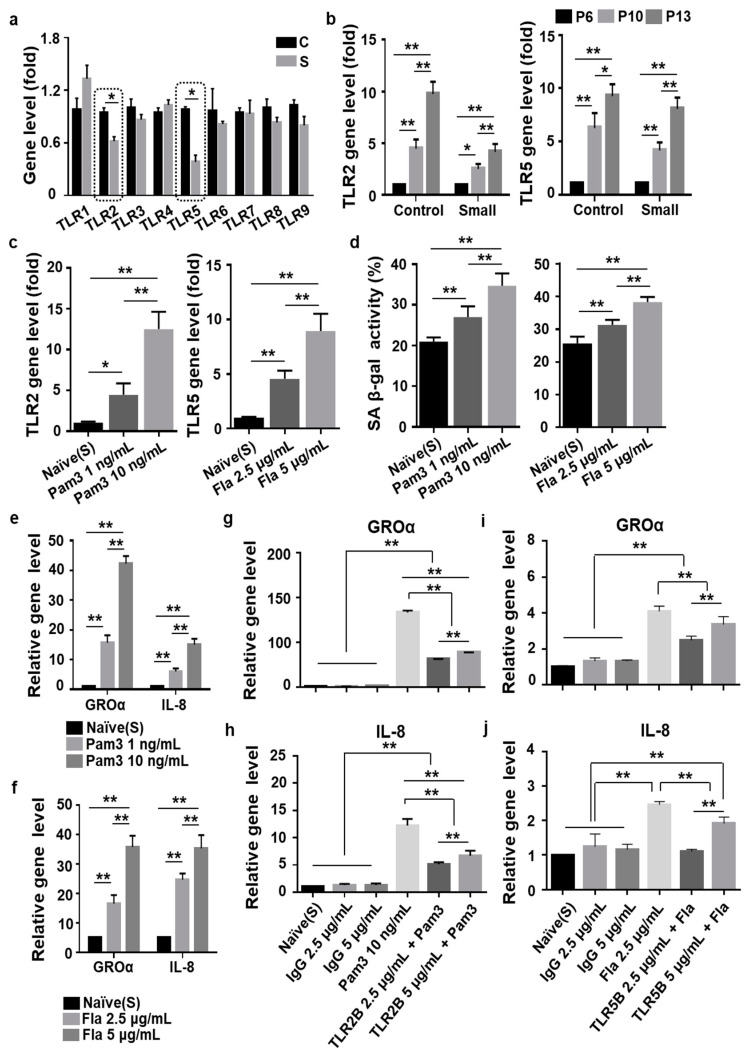
Toll-like receptor 2 (TLR2) and TLR5 activation induce the senescent phenotype in MSCs and were both decreased in small cells: (**a**) Quantification of gene expression levels of TLRs (1~9) in small cells or control cells at P13. Both TLR2 and TLR5 exhibited significantly lower expression in small cells compared to that in control cells (black box); (**b**) to confirm whether TLR2- (left panel) and TLR5-mediated signaling (right panel) in control and small cells is linked to the induction of cellular senescence; (**c**,**d**) We treated cells with different concentrations of Pam3 or Fla for 48 h; (**c**) quantification of gene expression levels of TLR2 (left panel) or TLR5 (right panel) in Pam3-treatment or Fla-treatment. Expression levels were normalized to those of **ß**-actin, with the expression level in naïve cells defined as 1; (**d**) cells were stained for SA β-gal, and activity was measured by counting the positively stained cells; (**e**,**f**) Gene expression of GROa (**e**) and IL-8 (**f**) in small cells treated with Pam3 or Fla was detected using qPCR; (**g**,**h**) Real-time PCR analysis of gene expression (GROa or IL-8) in small cells at P13 treated with isotype control IgG, TLR2 blocking (TLR2B), or TLR5 blocking (TLR5B) antibody in addition to Pam3 or Fla; (**e**–**h**) expression levels were normalized to those of β-actin, with the expression level in naïve cells defined as 1; (**a**–**h**) results are presented as mean ± SD, *n* = 3 per group. * *p* < 0.05. ** *p* < 0.01. C, control cells; S, small cells; P, passage; GROa, growth-regulated oncogene-alpha; IL-8, interleukin-8; toll-like receptor 2 blocking, TLR2B; toll-like receptor 5 blocking, TLR5B; SA β-gal staining; senescence-associated β-galactosidase staining; rhGROa, recombinant human GROa; rhIL-8, recombinant human IL-8.

It has been proposed that TLRs regulate SASP [32]. Herein, we assessed TLR (1–9) gene expression in control and small cells at P13 in order to investigate which of these receptors were associated with the delay of senescence observed in small cells. As a result, we identified two TLRs (2 and 5) that exhibited lower expression in small cells, as revealed using qPCR (Figure 5a). To further examine the association between these two TLRs (2 or 5) and cellular senescence, we analyzed the expression of TLR2 and TLR5 in both groups (control vs. small cells) during expansion at P6, P10, and P13. TLR2 and TLR5 levels were significantly upregulated by passaging in both groups. Further, cells of the two groups could be distinguished based on the rate of increase in TLR2 and TLR5 expression by passaging. The two groups exhibited a marked difference in fold increase from P6 to P13 (TLR2; Control vs. Small; 9.8-fold vs. 4.1-fold, *p* < 0.01; TLR5, Control vs. Small; 9.4-fold vs. 7.8-fold, *p* < 0.05, Figure 5b). To confirm the role of TLR2 or TLR5 in cellular senescence, we activated the small cells through treatment with ligands pam3CSK4 (Pam3; 1 or 10 ng/mL) or flagellin (Fla; 2.5 or 5 µg/mL) for 48 h. Pam3 or Fla treatment significantly upregulated TLR2 or TLR5 expression in a dose-dependent manner (Figure 5c). Based on the senescence phenotype, SA β-gal positive staining was significantly higher following Pam3 or Fla stimulation, increasing in a dose-dependent manner (Figure 5d and Figure S6). Next, we tested the effect of TLR2 or TLR5 stimulation on the expression of SASP-associated GROa and IL-8 in small cells. Compared with the untreated naïve cells, Pam3 or Fla5 treatment significantly upregulated both GROa and IL-8 expression in a dose-dependent manner (Figure 5e,f). To demonstrate that the two TLR receptors functionally contributed to cellular senescence in small MSCs, we inhibited TLR2 or TLR5 expression using a blocking antibody and prepared isotype (IgG)-treated cells as a control group. For example, cells from three different lots treated with Pam3 and a TLR2-blocking antibody exhibited senescent phenotypes (GROa and IL-8) to a much lesser extent compared to Pam3-stimulated cells (Figure 5g,h). We also observed significantly decreased secretion of GROa or IL-8 in cells treated with Fla and a TLR5-blocking blocking antibody when compared to Fla alone (Figure 5g,h). These data suggested that TLR2 and TLR5 induced senescence in MSCs by stimulating the secretion of SASP-related proteins. Furthermore, TLR2- and TLR5-mediated senescence was decreased in small cells.

## 4. Discussion

In vitro expansion of MSCs is necessary for their clinical use, but leads to cellular senescence [9,10,11]. Superior proliferation and delayed senescence were observed in small-sized MSCs compared to conventional cultures of heterogenous cells of various sizes. Among SASP-associated proteins involved in MSC senescence, the secretion of GROa and IL-8, which acted in a positive feedback loop with CXCR2, was low in small-sized MSCs. Furthermore, TLR2 and TLR5 were shown to regulate this signaling pathway.

Cellular senescence refers to cell cycle arrest under certain conditions and was first described by Hayflick using continuously cultured human fibroblasts [33,34]. The senescent phenotype is controlled by telomere length, but can also be induced by independent stimuli. Cellular senescence can be assessed through DNA damage and SA β-gal activity [18,19]. Furthermore, recent studies have reported that senescent cells have a unique secretome called SASP, which is characterized by inflammatory cytokines and metalloproteinases, greatly contributing to senescence [20].

Changes in signaling between cells that occur during senescence have also recently been described as inflammaging (a compound term for inflammation and aging), referring to the continuous inflammatory effect of senescent cells on their surroundings [35]. In addition, this suggests that the part of MSCs senescence work as a key player for beginning and inflammatory process and as an effective treatment for anti-aging strategies, however there is still unexplored region remaining to be elucidated, which contains the main mechanism that might contribute to inflammaging progression. [36].

According to the International Society for Cell and Gene Therapy (ISCT), MSCs exhibit heterogeneous properties depending on the donor, tissue of origin, and methods of isolation [37]. For example, in symmetric cell division, the parent cell divides into two daughter cells with identical morphology and characteristics as the parent cell [22]. In contrast, asymmetric cell division results in a self-renewable daughter cell and a non-dividing cell [22]. This causes pre-senescence in some cells during subculture, leading to heterogeneous characteristics of the population. Such characteristics affect cell proliferation, morphology, immunophenotype, and multipotency [38]. Colter, who classified MSCs into different sizes, demonstrated that small-sized MSCs had a faster proliferation rate compared to large MSCs [39]. Similarly, in our previous study, MSCs isolated from bone marrow, adipose tissue, and UCB varied in size, with small-sized cells exhibiting a higher proliferation rate and delayed senescence [26]. In addition, we demonstrated a main role of small cells in potentially enhancing the efficacy of MSCs transplantation [26]. However, the specific mechanism of delayed senescence in small-sized MSCs remains unknown.

Here, the rate of senescence was compared between cells in the control group (heterogenous) that were not sorted and small cells. In the control group at P13, more than 25 ± 6% of the cells were SA β-gal-positive. In contrast, only 8 ± 3% of the small cells were SA β-gal-positive, and the difference between the two groups was significant. Additionally, an inflammatory cytokine array for senescence-related proteins revealed that the levels of secreted IL-8 and GROa were significantly lower in small cells. In particular, the secretion of these two proteins was already lower in small cells at P6, as was the fold increase in secretion from P6 to P13 when compared to that in control cells. CXCR2 is a cognate receptor for IL-8 and GROa, and its expression increased over time as MSCs were sub-cultured. As expected, CXCR2 expression was lower in small cells than in the heterogeneous cells. It has been reported that SASP induced cells exert their effects not only locally, but also modulate their microenvironment via multiple regulatory routes, including (i) autocrine signaling by cell-of-origin, (ii) paracrine signaling by neighboring or surrounding cells, and (iii) positive feedback loop via autocrine/paracrine signaling [22]. The current study demonstrated that the main SASP-associated proteins (IL-8 and GROa) formed a positive loop with CXCR2 through autocrine/paracrine feedback during UCB-MSC senescence. Additionally, recombinant protein treatment and siRNA experiments demonstrated that the positive loop was less active in small-sized cells. Furthermore, it has been suggested that large-sized senescent neighboring cells induce senescence in healthy cells within a heterogeneous population. In contrast, small cells, which are relatively homogeneous, seem to play the role of “good” neighbors, delaying the rate of senescence. Similar to the current findings, previous studies have shown that IL-8 and GROa lead to enhanced senescence in fibroblasts through autocrine and paracrine action via CXCR2, regulating the DDR via Rb/p16 and p53/21 signaling [40,41].

TLRs are the major receptors of the innate immune system. When antigens from the external environment invade, TLRs recognize pathogen-associated molecular patterns (PAMPs), structures that are common among microorganisms [42]. Each of the 10 TLRs identified in humans recognizes specific ligands and plays an important role in the early stages of the immune response [24,25,42]. TLRs are known for their roles in cells of the innate immune response, including neutrophils and macrophages, as well as adaptive immune cells and even non-immune cells [43]. In a model of senescence, stimulation with lipopolysaccharide affected the secretion of inflammatory TNFα and anti-inflammatory IL-10 by macrophages isolated from the model, in addition to decreasing the expression of TLR2 and TLR4 [40]. These findings indicated that a decline in the function of senescent cells leads to the inactivation of TLRs. TLRs (TLR1–9) are expressed on human MSCs, and differences in the expression levels are observed depending on the tissue source [25,44]. During cellular senescence, persistent activation of TLR signaling was observed in MSCs, controlling the excessive production of inflammatory cytokines [44]. Based on this observation, it was hypothesized that TLR signaling is a driver of senescence in small-sized MSCs. Thus, TLR expression was compared between heterogeneous and small cells. The expression of TLR2 and TLR5 was significantly lower in small-cells. During subculture, the increase in TLR2 and TLR5 expression levels was also lower in small cells than in heterogeneous MSCs, which was similar to the trend observed for IL-8, GROa, and CXCR2. Additionally, treatment of small-sized MSCs with TLR2 and TLR5 ligands at various concentrations resulted in a significant increase in senescence markers such as SA β-gal activity and SASP-related protein levels (IL-8 and GROa). When the cells were treated with a blocking antibody in order to inhibit TLR2 and TLR5 receptors, SASP secretion was significantly reduced. These results demonstrate that GROa and IL-8 signaling via TLR2 and TLR5 is delayed in small-sized MSCs, leading to delayed cellular senescence. We previously reported TLR3 was upregulated in senescent MSCs and could trigger senescence via Janus kinase 1 (JAK1) signaling, a major factor in senescent cells. Importantly, the process was driven by the TLR3-dependent SASP autocrine loop wherein IFN-ß modulates TLR3 signaling [25]. Herein, we demonstrated that low activity of TLR2 and TLR5 regulated SASP secretion in small cells from MSCs. Studies have already reported that TLR2 and TLR5 are important factors in the regulation of cellular senescence [45,46]. According to Hari et al., innate immune sensing of senescence-related damage-associated molecular patterns (DAMPs) by TLR2 mediated the SASP and improved the cell cycle arrest program in oncogene induce senescence (OIS) [45]. Moreover, elevated IL-8 was accompanied by increased expression of TLR5 and increased levels of TLR5-mediated phosphorylation of MAPK p38 and ERK in the monocytes of elderly donors [43]. Assessment of the transcription patterns of senescent tissues revealed that overactivation of NF-кB was a key transcriptional characteristic of senescence [47,48]. Moreover, it is reported that pathogen-derived ligand induced TLR engagement and signal transduction lead to activate NF-кB signaling, and thereby to trigger the expression of proinflammatory and antiviral response genes [49]. Therefore, further studies are required to show that upstream mechanisms involving TLR2 and TLR5 regulate NF-кB activity, thereby increasing SASP cells.

## 5. Conclusions

In conclusion, SASP signaling via a positive loop between GROa, IL-8, and receptor CXCR2 was decreased in small cells from UCB-MSCs, leading to delayed cellular senescence in repetitive subcultures. Additionally, TLR2 and TLR5 activation was shown to regulate this process. The current findings may help enhance stem cell therapy for the treatment of otherwise incurable diseases.

## Data Availability

All data are included in the paper. There are no databases associated with this manuscript.

## Supplementary Materials

Supplementary Materials can be found at https://www.mdpi.com/2073-4409/10/1/63/s1. Figure S1: UCB-MSCs were isolated based on cell size using a 10-µm filter system at P2; Figure S2: Naïve MSCs were treated with rhGROa or rhIL-8 at P13; Figure S3: Silencing of GROa, IL-8, or CXCR2 expression in small MSCs at P13; Figure S4: Small MSCs were transfected with scramble siRNA (siCon MSC) or CXCR2 siRNA (siCXCR) at P13; Figure S5: Heterogenous cells were transfected with scramble siRNA (siCon MSC) or CXCR2 siRNA (siCXCR) at P13; Figure S6: TLR (2 or 5) activation triggers the senescent phenotype in small cells at P13; Supplemental Table S1: Basic information of UCB-MSCs characterization; Supplemental Table S2: For the human cytokine array panel; Supplemental Table S3: Sequences of primers used for indicated target genes.

## Abbreviations

UCB-MSC	Umbilical cord blood-derived mesenchymal stem cell
SASP	Senescence-associated secretory phenotype
GROa	Growth-regulated oncogene-alpha
IL-8	Interleukin-8
CXCR2	C-X-C motif chemokine receptor 2
TLR2	Toll-like receptor 2
TLR5	Toll-like receptor 5
PD	Population doubling
SA β-gal	senescence-associated beta-galactosidase
