# Supplementary material for: Senescence-Associated Secretory Phenotype Suppression Mediated by Small-Sized Mesenchymal Stem Cells Delays Cellular Senescence through TLR2 and TLR5 Signaling
Source: Cells. 2021 Jan 3;10(1):63. doi: 10.3390/cells10010063 (PMC7824096; doi:10.3390/cells10010063)
Supplement: Supplementary file 1 [file cells-10-00063-s001.pdf]

Supplementary Materials:

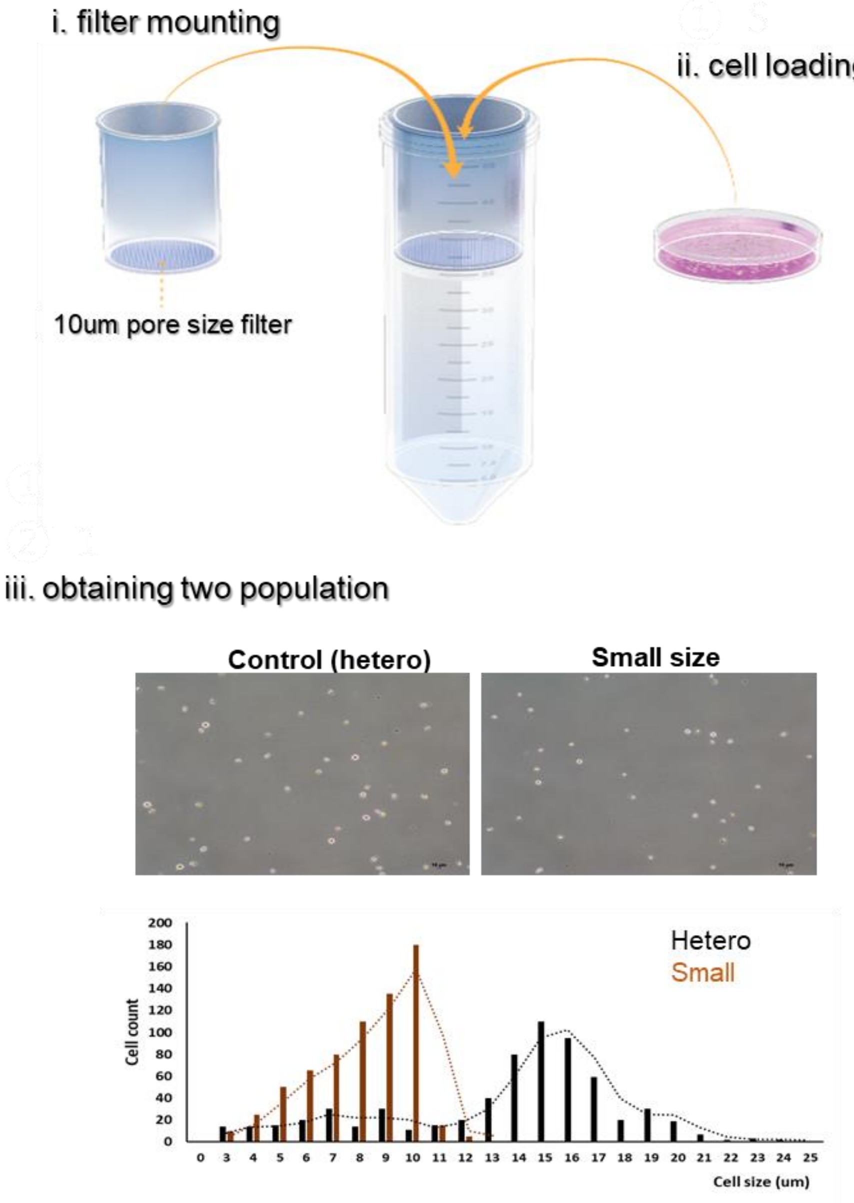

Figure S1. UCB-MSCs were isolated based on cell size using a 10 μm filter system at P2: (**i**) filter mounting: the filtration membrane was inserted in 50 mL culture tube. (**ii**) cell loading: cell was loaded on filtration membrane tube. (**iii**) Obtaining two populations: heterogeneous cell and filtered small cells were obtained. Cells size was measured under a microscope following isolation. Scale bar = 10 μm. P, passage; MSCs, mesenchymal stem cells.

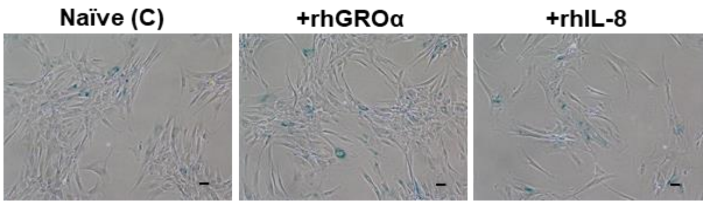

Figure S2. Naïve MSCs were treated with rhGROα or rhIL-8 at P13. Senescence was evaluated based on microscopic morphology assessment and SA β-gal staining. Cells treated with rhGROα or rhIL-8 showed strong positive staining. Scale bar = 10 μm. rhGROα; recombinant human GROα, rhIL-8; recombinant human IL-8, SA β-gal; senescence associated beta-galactosidase.

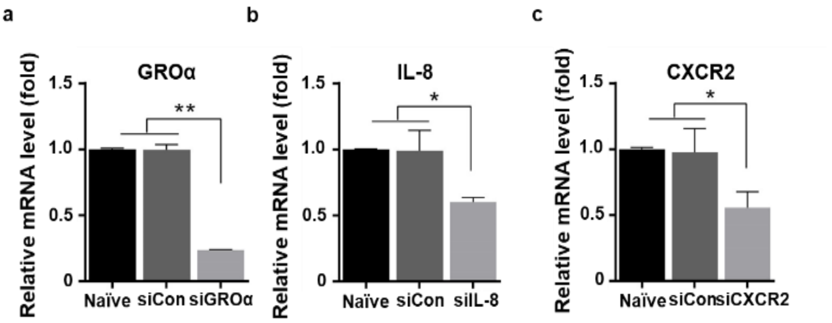

Figure S3. Silencing of GROα, IL-8, or CXCR2 expression in small MSCs at P13. Cell were transfected with scramble siRNA (siConMSC) or CXCR2 siRNA (siCXCR) at P13: (**a**) GROα, (**b**) IL-8, and (**c**) CXCR2 gene expression decreased in target siRNA-treated cells compared to naïve or siCon-treated cells. Downregulated expression of these three genes was confirmed by qPCR. The expression levels of all genes were normalized to those of β-actin in naïve cells, which was defined as 1-fold expression (mean ± SD, n = 3; * < 0.05, ** p < 0.01). IL-8, interleukin-8; GROα, growth-related oncogene-alpha; CXCR2, C-X-C motif chemokine receptor 2.

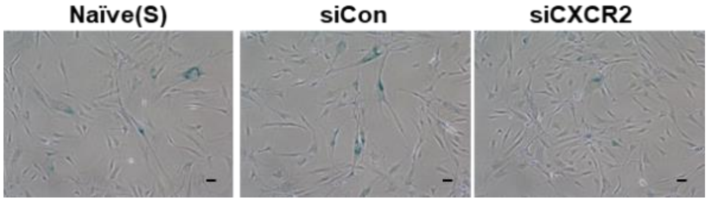

Figure S4. Small MSCs were transfected with scramble siRNA (siCon) or CXCR2 siRNA (siCXCR) at P13. Senescence was evaluated based on morphological assesssment using microscopy and SA β -gal staining. Silencing of CXCR2 inhibited positive staining. Scale bar = 10 μm. siCon, scramble siRNA; siCXCR2, CXCR2 siRNA; SA β-gal, senescence associated beta-galactosidase.

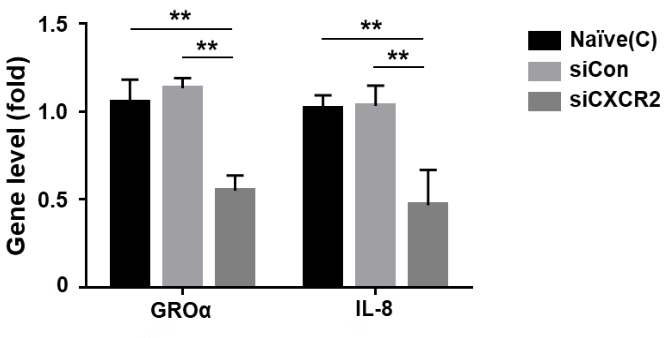

Figure S5. Heterogenous cells were transfected with scramble siRNA (siCon) or CXCR2 siRNA (siCXCR) at P13. GROα and IL-8 expression was quantified using qPCR. The expression levels of all genes are normalized to those of β-actin in naïve cells, which is defined as 1-fold expression (mean ± SD, n = 3; ** p< 0.01). IL-8, interleukin-8; GROα, growth-related oncogene-alpha; siCon, scramble siRNA; siCXCR2, CXCR2 siRNA.

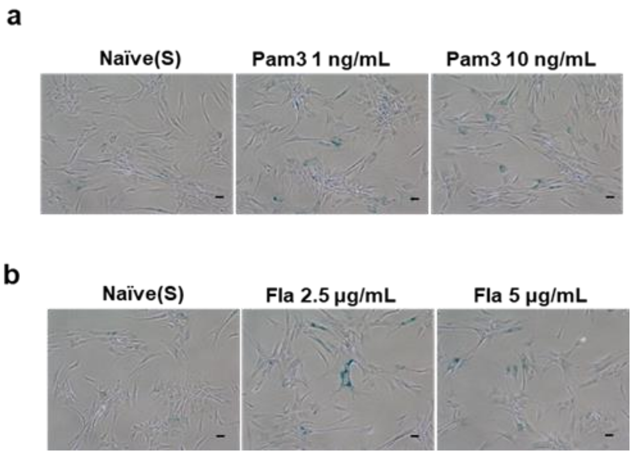

Figure S6. TLR (2 or 5) activation triggers the senescent phenotype in small cells at P13: (**a**) Senescnce was evaluated by microscopic morphology assessment and SA ß-gal staining of Pam3-treated cells. (**b**) Senescence was evaluated by microscopic morphology assessment and SA ß-gal staining of flagellin-treated cells. Pam3, pam3CSK4; Fla, flagellin; SA β-gal, senescence associated beta-galactosidase.

Supplemental Table 1. Basic information of UCB-MSCs characterization.

UCB-MSCs	Surface marker		Differentiation
	Positive	Negative	
#1	Pass	Pass	Pass
#2	Pass	Pass	Pass
#3	Pass	Pass	Pass

UCBs were isolated from three independent donors (UCB #1 to 3). MSC characterization was performed by assessing MSC surface marker expression and MSC differentiation capacity (positive: CD29, CD73, CD90, CD105, and CD166 ≥ 80%; negative: CD14, CD45 ≤ 1.0%; differentiation: osteogenic, chondrogenic, adipogenic).

Supplemental Table 2. For the human cytokine array panel

Coordinate	Target
A1, A2	Reference Spot
A3, A4	C/C5a
A5, A6	CD40 Ligand
A7, A8	G-CSF
A9, A10	GM-CSF
A11, A12	GROα
A13, A14	I-309
A15, A16	sICAM-1
A17, A18	IFN-λ
A19, A20	Reference Spot
B3, B4	IL-1α

B5, B6	IL-1ß
B7, B8	IL-1rα
B9, B10	IL-2
B11, B12	IL-4
B13, B14	IL-5
B15, B16	IL-6
B17, B18	IL-8
C3, C4	IL-10
C5, C6	IL-12p70
C7, C8	IL-13
C9, C10	IL-16
C11, C12	IL-17
C13, C14	IL-17E
C15, C16	IL-23
C17, C18	IL-27
D3, D4	IL-32α
D5, D6	IP-10
D7, D8	I-TAC
D9, D10	MCP-1
D11, D12	MIF
D13, D14	MIP-1α
D15, D16	MIP-1ß
D17, D18	Serpin E1
E1, E2	Reference Spot
E3, E4	RANTES
E5, E6	SDF-1
E7, E8	TNF-α
E9, E10	sTREM-1
E19, E20	Negative Control

Supplemental Table 3. Sequences of primers used for indicated target genes.

**Table Gene.**	**Primer Sequence (5′-3′)**
Scramble siRNA	UGGUUUACAUGUCGACUAA<br>UGGUUUACAUGUUGUGUGA<br>UGGUUUACAUGUUUUCUGA<br>UGGUUUACAUGUUUUCCUA
GROα siRNA	GAUGCUGAACAGUGACAAA<br>CGGAAAGCUUGCCUCAAUC<br>UUACAGUGUUUCUGGCUUA<br>GCUGGCGGAUCCAAGCAAA
IL-8 siRNA	ACUAAGAGUGGUCGAAGAA<br>GCACAGCAGCAGAUCGAUU<br>CAUAGAAGGACACGUGGUA<br>GAUACAGGCUCCAGUCAUA
CXCR2 siRNA	UCUAAGACCUCCUGCCUAA<br>GAAGGACCGUCUACUCAUC<br>CCUCAAGAUUCUAGCUAUA<br>GAGGACAUGGGCAACAAUA
